# Supplementary material for: Associations of PM2.5 and its components with term preterm rupture of membranes: a retrospective study
Source: PeerJ. 2025 Jan 31;13:e18886. doi: 10.7717/peerj.18886 (PMC11789663; doi:10.7717/peerj.18886)
Supplement: Supplemental Information 1 [file peerj-13-18886-s001.docx]

Table S1 Spearman correlation coefficients for mean daily concentrations of PM_2.5_ and its components

|  | PM_2.5_ | SO_4_^2-^ | NO_3_^-^ | NH_4_^+^ | OM | BC |
| --- | --- | --- | --- | --- | --- | --- |
| PM_2.5_ | 1.000 |  |  |  |  |  |
| SO_4_^2-^ | 0.973 | 1.000 |  |  |  |  |
| NO_3_^-^ | 0.933 | 0.853 | 1.000 |  |  |  |
| NH_4_^+^ | 0.964 | 0.905 | 0.990 | 1.000 |  |  |
| OM | 0.990 | 0.984 | 0.898 | 0.941 | 1.000 |  |
| BC | 0.969 | 0.988 | 0.839 | 0.892 | 0.989 | 1.000 |

*PM_2.5_*, particulate matter with aerodynamic diameter of ≤2.5μm. *SO_4_^2-^*, sulfate. *NO_3_^-^*, nitrate. *NH_4_^+^*, ammonium. *OM*, organic matter. *BC*, black carbon.

All correlations are significant at *P* <0.001
