# Supplementary material for: Associations of PM2.5 and its components with term preterm rupture of membranes: a retrospective study
Source: PeerJ. 2025 Jan 31;13:e18886. doi: 10.7717/peerj.18886 (PMC11789663; doi:10.7717/peerj.18886)
Supplement: Supplemental Information 2 [file peerj-13-18886-s002.docx]

**STROBE Statement—checklist of items that should be included in reports of observational studies**

|  | **Item No.** | **Recommendation** | **Page No.** | **Relevant text from ttmanuscript** |
| --- | --- | --- | --- | --- |
| **Title and abstract** | 1 | (*a*) Indicate the study’s design with a commonly used term in the title or the abstract | 1 | A retrospective study |
|  |  | (*b*) Provide in the abstract an informative and balanced summary of what was done and what was found | 2 | Background  There is evidence that fine particulate matter (PM2.5) exposure is associated with premature rupture of membranes (PROM); however, studies of its effect on term PROM (TPROM) are limited, and the results are inconsistent.  Objective  This study aimed to investigate the association between exposure to PM2.5 and its components and the risk of TPROM.  Methods  From 2018 to 2022, we collected delivery data from pregnant women in Guangzhou. Using 1:1 case matching, we included 1,216 TPROM cases and 1,216 controls. PM2.5 and its component concentrations were obtained from Tracking Air Pollution in China. The time-varying mean concentration method was used to estimate exposure to PM2.5 and its components during different trimesters. Cox proportional hazards models were used to calculate hazard ratios (HRs) and 95% confidence intervals (CIs) to evaluate the associations of exposure to PM2.5 and its components with the risk of TPROM.  Results  The incidence of TPROM in this study was 19.66%. After adjusting for potential confounders, statistically significant associations were found between TPROM and exposure to PM2.5, NO3-, NH4+, and BC during the second trimester and between TPROM and exposure to PM2.5, SO42-, and BC during the third trimester. Specifically, the IQR3 and IQR4 of SO42- exposure during the third trimester increased the risk of TPROM by 18% (95% CIs: 1.01−1.39) and 18% (95% CIs: 1.01−1.39), respectively. A nonlinear relationship was observed between exposure to PM2.5, SO42-, NH4+, and OM during the second trimester and the risk of TPROM. No significant interactions were found between PM2.5 and its components with TPROM across various subgroups.  Conclusion  Our findings indicate significant associations between the risk of TPROM and exposure to PM2.5 and several of its components during pregnancy. Contribute to the literature on the associations of PM2.5 and its components with TPROM. |
| **Introduction** |  |  |  |  |
| Background/rationale | 2 | Explain the scientific background and rationale for the investigation being reported | 3 | Despite the widespread concern about preterm PROMs, TPROMs are also a pregnancy complication that should not be ignored, and their occurrence can lead to adverse outcomes such as perinatal death, increased rates of caesarean section, early-onset neonatal pneumonia, and neonatal sepsis.  Although the causes of PROM remain unclear, several factors have been implicated, including age, previous preterm birth, smoking, polyhydramnios, urinary and sexually transmitted infections, previous PROM, caesarean section and cervical incompetence. Exposure to fine particulate matter (PM2.5) increases the risk of PROM, according to the findings of recent investigations into the relationship between air pollutants and PROM |
| Objectives | 3 | State specific objectives, including any prespecified hypotheses | 4 | The aim of this study was to analyse data from pregnant women delivering at the Maternal and Children Health Care Hospital of Huadu in Guangzhou from 2018−2022, with a focus on the associations between exposure to PM2.5 and its components and TPROM during pregnancy. |
| **Methods** |  |  |  |  |
| Study design | 4 | Present key elements of study design early in the paper | 4 | This retrospective study involved pregnant women who delivered at the Maternal and Children Health Care Hospital of Huadu in Guangzhou from November 2018 to December 2022. Data were collected using the hospital’s electronic medical record management system. Relevant information, including age, occupation, ethnicity, blood type, delivery date, and clinical diagnosis, was obtained by reviewing the electronic medical records. Diagnoses were classified according to the ICD-10 codes. |
| Setting | 5 | Describe the setting, locations, and relevant dates, including periods of recruitment, exposure, follow-up, and data collection | 4 | This retrospective study involved pregnant women who delivered at the Maternal and Children Health Care Hospital of Huadu in Guangzhou from November 2018 to December 2022. Data were collected using the hospital’s electronic medical record management system  the spatially continuous, grid-based daily average concentrations of PM_2.5_ and its constituents from 2018−2022 from tracking air pollution (TAP) in China (<http://tapdata.org.cn/>). |
| Participants | 6 | (*a*) *Cohort study*—Give the eligibility criteria, and the sources and methods of selection of participants. Describe methods of follow-up  *Case-control study*—Give the eligibility criteria, and the sources and methods of case ascertainment and control selection. Give the rationale for the choice of cases and controls *Cross-sectional study*—Give the eligibility criteria, and the sources and methods of selection of participants | 4 | **Eligibility Criteria and Participant Selection Methods in Different Study Designs:**  **Cohort Study:**  The exclusion criteria for the study participants were as follows: (1) residing outside Guangzhou; (2) in vitro fertilisation; (3) pregnancy of twins; (4) diabetes before pregnancy; (5) hypertension before pregnancy; and (6) insufficient data |
|  |  | (*b*) *Cohort study*—For matched studies, give matching criteria and number of exposed and unexposed  *Case-control study*—For matched studies, give matching criteria and the number of controls per  case | 6 | This retrospective study used a case‒control design, with cases being TPROM patients and controls being non-TPROM patients. Patients and controls were matched 1:1 for age and last menstrual period (LMP) |
| Variables | 7 | Clearly define all outcomes, exposures, predictors, potential confounders, and effect modifiers.  Give diagnostic criteria, if applicable | 5-6 | The participants provided self-reported information regarding their ethnicity (such as Han, Hui, Miao, and Tujia), occupation category (such as employee, civil servant, professional, self-employed, farmer, and unemployed), marital status (married or divorced), and blood type (A, B, O, and AB), and the study focused on women who were pregnant and gave birth for the first time. For further analysis, ethnicity was reclassified into Han or other, occupation type was regrouped as employed, self-employed, or other, and infant weight was stratified on the basis of recorded birth weight into low birth weight (< 2500 g), normal birth weight (2500-4000 g), or macrosomia (> 4000 g). Hepatitis B virus status indicates that a subject is a carrier of HBV. Obesity during pregnancy was defined as a body mass index greater than or equal to 28.0 kg/m² during pregnancy. An adverse reproductive history refers to a history of previous poor pregnancy outcomes or obstetric complications.  TPROM was determined based on the clinical diagnoses in the electronic medical records system. The diagnosis of PROM is based on three specific indicators observed by the clinician during a sterile speculum examination: (1) accumulation of clear fluid in the posterior vaginal fornix or exudation of fluid from the cervical orifice; (2) alkaline pH of the cervicovaginal secretions, usually detected by a change in colour from yellow to blue on nitrozine paper; and/or (3) the appearance of fernlike patterns in the dry cervicovaginal secretions under the microscope |
| Data sources/ measurement | 8* | For each variable of interest, give sources of data and details of methods of assessment (measurement). Describe comparability of assessment methods if there is more than one group | 4-5 | PM_2.5_ and its constituents from 2018−2022 from tracking air pollution (TAP) in China (<http://tapdata.org.cn/>)  from the hospital’s electronic medical record system. The covariates were age, ethnicity, occupation, blood type, primary, anaemia, adverse reproductive history, uterine scar, hepatitis B virus (HBV) status, and obesity during pregnancy. |
| Bias | 9 | Describe any efforts to address potential sources of bias | 6 | Patients and controls were matched 1:1 for age and last menstrual period (LMP). This matching strategy aimed to ensure that all participants had the same starting point in early pregnancy, thereby placing each in a similar environment for most of their pregnancies. Once matching was complete, a Cox proportional hazards model was used to estimate hazard ratios (HRs) and 95% confidence intervals (CIs) to assess the associations of exposure to PM_2.5_ and its components with the risk of TPROM.  used Cox proportional hazards model alongside restricted cubic splines curves to assess the relationship between exposure to PM_2.5_ and its constituents and the risk of TPROM |
| Study size | 10 | Explain how the study size was arrived at | 6 | Cox proportional hazards model was used to estimate hazard ratios (HRs) and 95% confidence intervals (CIs) to assess the associations of exposure to PM_2.5_ and its components with the risk of TPROM, controlling for potential confounders, including age, occupation, blood group, ethnicity, parity, HBV status, obesity during pregnancy, adverse reproductive history, and uterine scar |
| Continued on next page |  |  |  |  |

| Quantitative variables | 11 | Explain how quantitative variables were handled in the analyses. If applicable, describe which groupings were chosen and why.  ***Page 6***  Cox proportional hazards model was used to estimate hazard ratios (HRs) and 95% confidence intervals (CIs) to assess the associations of exposure to PM2.5 and its components with the risk of TPROM, aiming to discern statistically significant associations  **Page 7**  Quantitative variables including PM_2.5_, SO_4_^2-^, NO_3_^-^, NH_4_^+^, OM and BC were assessed using Spearman correlation to evaluate statistical dependence and potential predictive relationships. |
| --- | --- | --- |
| Statistical methods | 12 | 1. Describe all statistical methods, including those used to control for confounding   **Page 6**  Cox proportional hazards model was used to estimate hazard ratios (HRs) and 95% confidence intervals (CIs) to assess the associations of exposure to PM_2.5_ and its components with the risk of TPROM, controlling for potential confounders, including age, occupation, blood group, ethnicity, parity, HBV status, obesity during pregnancy, adverse reproductive history, and uterine scar.  Cox proportional hazards model alongside restricted cubic splines curves to assess the relationship between exposure to PM_2.5_ and its constituents and the risk of TPROM. The reference value, established at the 10th percentile with an HR of 1, was compared using knots positioned at the 5th, 35th, 65th, and 95th percentiles of the concentration levels of PM_2.5_ and its components. |
|  |  | 1. Describe any methods used to examine subgroups and interactions   **Page 6**  In addition, interaction analyses were performed to explore possible interactions between exposure to air pollutants during pregnancy and these confounders |
|  |  | (*c*) Explain how missing data were addressed *NA* |
|  |  | (*d*) *Cohort study*—If applicable, explain how loss to follow-up was addressed *NA*  *Case-control study*—If applicable, explain how the matching of cases and controls was addressed *Cross-sectional study*—If applicable, describe analytical methods taking account of the sampling strategy *NA* |
|  |  | (*e*) Describe any sensitivity analyses: ***The study did not conduct sensitivity analyses to assess the robustness of the results*** |
| **Results** |  |  |
| Participants | 13* | 1. Report numbers of individuals at each stage of study—eg numbers potentially eligible, examined for eligibility, confirmed eligible, included in the study, completing follow-up, and analysed   **Page 6**  In a sample of 15,633 cases, 19.66% of full-term pregnant women experienced TPROM. Using 1:1 case matching, we included 1,216 TPROM cases and 1,216 controls |
|  |  | (b) Give reasons for non-participation at each stage  ***NA*** |
|  |  | (c) Consider use of a flow diagram  Figure 1 |
| Descriptive data | 14* | 1. Give characteristics of study participants (eg demographic, clinical, social) and information on exposures and potential confounders   **Page 6**  Table 1 After matching, the Z-values or χ^2^-values for age, ethnicity, occupation, primary, anaemia, adverse reproductive history, uterine scar, HBV status, infant weight, and pollution concentration all decreased to varying degrees. Specifically, age, race, occupation and adverse obstetric history changed significantly before and after matching, with p-values of 0.025, 0.016, <0.001 and 0.017 before matching and 1,000, 0.169, 0.777 and 0.205 after matching, respectively |
|  |  | (b) Indicate the number of participants with missing data for each variable of interest ***NA*** |
|  |  | (c) *Cohort study*—Summarise follow-up time (eg, average and total amount)  **This retrospective study involved pregnant women who delivered at the Maternal and Children Health Care Hospital of Huadu in Guangzhou from November 2018 to December 2022** |
| Outcome data | 15* | *Cohort study*—Report numbers of outcome events or summary measures over time  ***NA*** |
|  |  | *Case-control study—*Report numbers in each exposure category, or summary measures of exposure  **Patients and controls were matched 1:1 for age and last menstrual period (LMP).**  **Using 1:1 case matching, we included 1,216 TPROM cases and 1,216 controls** |
|  |  | *Cross-sectional study—*Report numbers of outcome events or summary measures  **NA** |
| Main results | 16 | 1. Give unadjusted estimates and, if applicable, confounder-adjusted estimates and their precision (eg, 95% confidence interval). Make clear which confounders were adjusted for and why they were included   **We found that exposure to PM_2.5_, SO_4_^2-^, NO_3_^-^, NH_4_^+^, and BC was significantly associated with an increased risk of TPROM** |
|  |  | 1. Report category boundaries when continuous variables were categorized   ***The study did not categorize continuous variables but analyzed them in their continuous form*** |
|  |  | (*c*) If relevant, consider translating estimates of relative risk into absolute risk for a meaningful time **NA** |

Continued on next page

| Other analyses | 17 | Report other analyses done—eg analyses of subgroups and interactions, and sensitivity analyses  **Page 7-8**  In subgroup analyses after adjustment for confounders, we observed no significant interactions between exposure to PM_2.5_, SO_4_^2-^, NO_3_^-^, NH_4_^+^, OM, or BC and TPROM during pregnancy in terms of age, occupation, ethnicity, parity, HBV status, adverse reproductive history, or uterine scar subgroup |
| --- | --- | --- |
| **Discussion** |  |  |
| Key results | 18 | Summarise key results with reference to study objectives   - We found that exposure to PM_2.5_, SO_4_^2-^, NO_3_^-^, NH_4_^+^, and BC was significantly associated with an increased risk of TPROM. - We conducted subgroup analyses by age, ethnicity, occupation, blood type, parity, adverse reproductive history, and uterine scarring, we did not observe any significant interactions between exposure to PM_2.5_ and its components and TPROM |
| Limitations | 19 | Discuss limitations of the study, taking into account sources of potential bias or imprecision. Discuss both direction and magnitude of any potential bias  ①First, the exposure assessment was based on the average exposure level at the home address, without accounting for individual exposure variations at different locations, such as daily activity spaces and workplaces. Therefore, the values may not accurately reflect the true exposure levels of individuals and could lead to biased results.  ②Second, the analysis did not include certain health behaviours and lifestyle factors, such as smoking or cocaine use during pregnancy, as covariates, which increase the risk of PROM. Other factors, such as diet, exercise, and weight management, could also influence the results. Although age and last menstrual period were used as matching criteria to reduce confounding effects from age and gestational age, differences in gestational age might have biased the results.  ③Finally, there are limitations regarding sample representativeness; the participants were primarily from Guangzhou, which may restrict the generalisability and applicability of the findings. The regional and socioeconomic backgrounds of the participants may not be broadly representative, affecting the applicability of the results. |
| Interpretation | 20 | Give a cautious overall interpretation of results considering objectives, limitations, multiplicity of analyses, results from similar studies, and other relevant evidence   - The results of this study differ from those of previous investigations. In this study, exposure to PM_2.5_ during the second and third trimesters of pregnancy increased the risk of TPROM. First, differences in geographical regions and environmental conditions between studies could lead to differences in the composition and concentration of PM_2.5_, which could affect the risk of PROM. Second, differences in study methods and designs, such as sample size, exposure assessment methods and analytical techniques, could affect the accuracy of the results. Finally, differences in the lifestyle, health status and genetic background of pregnant women may lead to different responses to PM_2.5_ exposure. - he results of these works differ slightly from those of this study, in which prenatal exposure to SO_4_^2-^, NO_3_^-^, NH_4_^+^, and BC was significantly associated with an increased risk of TPROM. Inconsistencies in results may stem from multiple factors. First, the precision of exposure assessment may be affected by the use of different assessment methods, such as monitoring station data and satellite remote-sensing data, thereby influencing the estimation of the relationship between exposure and health. Second, the biological activities of the components of PM_2.5_ differ, and their direct effects on membranes and indirect effects on maternal physiology can vary depending on region and environmental conditions. Third, discrepancies in the level of control over confounding factors in studies also contribute to the variations in results***.*** |
| Generalisability | 21 | Discuss the generalisability (external validity) of the study results   - pregnant women should therefore minimise outdoor activities during peak pollution periods and may use air purifiers to reduce indoor PM_2.5_ levels. If outdoor activities in highly polluted areas are necessary, masks and other personal protective equipment should be worn to reduce the risk of inhaling harmful particulate matter. In addition, government policies can achieve long-term improvements in air quality through a range of measures, including increasing urban green spaces; strictly enforcing vehicle emission standards; restricting highly polluting industrial activities; and promoting the use of clean energy. These integrated strategies not only help improve environmental quality in general but also effectively reduce the risk of PROM in pregnant women. |
| **Other information** | | |
| Funding | 22 | Give the source of funding and the role of the funders for the present study and, if applicable, for the original study on which the present article is based   - **No funding.** |

*Give information separately for cases and controls in case-control studies and, if applicable, for exposed and unexposed groups in cohort and cross-sectional studies.

**Note:** An Explanation and Elaboration article discusses each checklist item and gives methodological background and published examples of transparent reporting. The STROBE checklist is best used in conjunction with this article (freely available on the Web sites of PLoS Medicine at [http://www.plosmedicine.org/,](http://www.plosmedicine.org/) Annals of Internal Medicine at [http://www.annals.org/,](http://www.annals.org/) and Epidemiology at [http://www.epidem.com/).](http://www.epidem.com/)) Information on the STROBE Initiative is available at [www.strobe-statement.org.](http://www.strobe-statement.org/)
